# Inhibition of the angiogenesis and growth of Aloin in human colorectal cancer *in vitro and in vivo*

**DOI:** 10.1186/1475-2867-13-69

**Published:** 2013-07-12

**Authors:** Qin Pan, Hongming Pan, Haizhou Lou, Yinghua Xu, Lu Tian

**Affiliations:** 1 Department of Medical Oncology, Sir Run Run Shaw Hospital, College of Medicine, Zhejiang University, 3 Qingchun East Road, Hangzhou 310016, China; 2 The First Affiliated Hospital, College of Medicine, Zhejiang University, 79 Qingchun Road, Hangzhou 310003, China

**Keywords:** Aloin, Angiogenesis, Tumor growth, Colorectal cancer, STAT3

## Abstract

**Background:**

Angiogenesis has been an attractive target for drug therapy. Aloin (AL), an natural compound derived from *Aloe barbadensis* Miller leaves, has been shown to possess anti-cancer potential activities. However, its roles in tumor angiogenesis and the involved molecular mechanism are unknown.

**Method:**

To evaluate the antiangiogenic and anticancer activities of AL, endothelial cell scratch, modified Boyden chamber inserts and tube formation assays were done in HUVECs, and MTT and Live-Dead assays were used to determine the proliferation inhibition and apoptosis induction of colorectal cancer cells *in vitro.* The inhibition effects of AL were further confirmed by a mouse xenograft model *in vivo*. The expression levels of STAT3 signaling pathway and that mediated-target genes were measured in HUVECs and SW620 cells by Western blots.

**Results:**

Here, we demonstrated that AL significantly inhibited HUVECs proliferation, migration and tube formation *in vitro*. Western blotting showed that AL suppressed activation of VEGF receptor (VEGFR) 2 and STAT3 phosphorylation in endothelial cells. In addition, the constitutively activated STAT3 protein, and the expression of STAT3-regulated antiapoptotic (Bcl-xL), proliferative (c-Myc), and angiogenic (VEGF) proteins were also down-regulated in response to AL in human SW620 cancer cells. Consistent with the above findings, AL inhibited tumor cell viability and induced cell apoptosis *in vitro*, and substantially reduced tumor volumes and weight in *vivo* mouse xenografts, without obviously toxicity.

**Conclusion:**

Our studies provided the first evidence that AL may inhibit tumor angiogenesis and growth *via* blocking STAT3 activation, with the potential of a drug candidate for cancer therapy.

## Introduction

Colorectal cancer (CRC) is the most common cause of cancer-related mortality, with an estimated over 1.2 million new diagnoses and 608,700 deaths worldwide [1]. Outcomes for patients with advanced CRC remain poor, with the median survival of still less than 20 months [2]. The large number of cases and the continued poor survival rates in CRC underscores the need for new therapy strategy.

The ability of tumors to progress to more malignant phenotypes is dependent on the tumor microenvironment. Angiogenesis, the development of new blood vessels from preexisting vascular bed, plays essential roles in tumor growth, maintenance, and metastasis [3]. At present, inhibition of tumor angiogenesis is considered as a promising strategy for the treatment of cancer [4]. Vascular endothelial growth factor (VEGF) is a potent pro-angiogenic factor crucial for tumor vascular development [5]. Vascular endothelial growth factor receptor 2 (VEGFR2) is the primary receptor of VEGF and the major mediator of VEGF-induced angiogenesis pathways [6].When resting endothelial cells are activated, VEGFR2 signaling activates a number of downstream mediators and allows cells to proliferate, migrate, invasive and finally differentiate to form capillary-like structures [7]. Recently studies showed that among VEGFR2-mediated signaling, especially signal transducer and activator of transcription 3 (STAT3), has been strongly implicated to be the hallmark of a wide variety of human malignancies and is commonly associated with a worse prognosis [8,9].

STAT3 belongs to a member of latent self-signaling transcription factors in cytoplasm activated by certain cytokines (e.g., IL-6) and growth factors (e.g., VEGF). Upon activation, STAT3 homodimerizes and translocates to the nuclear to subsequently modulates the transcription of responsive genes encoding apoptosis inhibitors (e.g., Bcl-xL, Bcl-2), proliferation regulatory proteins (e.g., cyclin D1, c-myc), and inducers of angiogenesis (e.g., VEGF) [10], which were involved with cell proliferation, survival, differentiation, apoptosis, metastasis, angiogenesis, host immune evasion, and drug resistance [11,12]. More recently, there is ample evidence in the literatures that interference of constitutive STAT3 signaling successfully results in an inhibition of growth and the induction of apoptosis in tumors [10,13]. Given the oncogenic function of STAT3 and promise of inhibiting it, directly targeting STAT3 signaling cascade has been an attractive therapeutic target for drug intervention to treat cancer.

**Figure 1:**
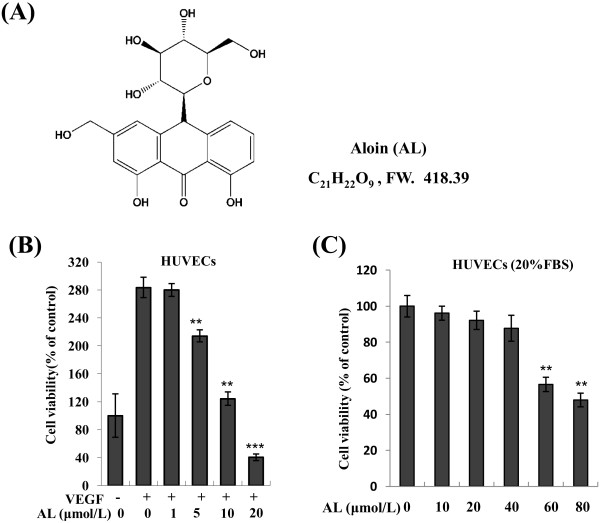
**Effect of AL on endothelial cell proliferation *in vitro*. A**, the structure of Aloin. **B**, treatment with AL significantly inhibited VEGF-induced HUVECs proliferation in dose-dependent manner as described in “Materials and methods.” **C**, effects of AL on HUVECs viability under normal culture condition. Cell viability was quantified by MTT assay. The bars represent triplicate analysis.**, *P* < 0.01; ***, *P* < 0.001 *vs.* control.

Recently agents that inhibited angiogenesis and targeted STAT3 have been identified from plants, with little side effects [13-15]. Aloin (AL; C_21_H_22_O_9_; Figure 1A), a natural bioactive anthracycline, derived from *Aloe barnadensis* Miller leaves (also called Aloin A or Barbaloin or10-β-D-Glucopyranosyl-1,8-dihydroxy-3-(hydroxymethyl)-9(10H)-anthracenone;), which was reported to show pharmacological effects, such as anti-inflammatory, antimicrobial, antioxidant activities, anti-virus and anti-cancer potential [16]. Studies showed that AL was able to induce cell cycle arrest and apoptosis in various human cancer cells, including breast [17], ovarian [18], uterine carcinoma [19], B16-F10 murine melanoma [16], and human Jurkat T lymphocytes cells [20]. Moreover, it was also reported to show wonderful healing and softening properties [21,22], suggesting the potential role of AL in antiangiogenesis. Therefore, these prompted us to evaluate the antiangiogenic and anticancer activities of AL and to fully elucidate its molecular mechanisms with special focus on STAT3 signaling pathway in human umbilical vein endothelial cells (HUVECs) and colorectal cancer cells. In the present study, we report our findings on human colorectal cancer growth suppressive activities of AL, its efficacy in inhibiting constitutive STAT3 signaling *in vitro*, and the effects on the processes of tumor angiogenesis and growth *in vivo*.

## Materials and methods

### Reagents

AL (purity > 97%) was purchased from Sigma (St. Louis, MO). A 20 mmol/L solution of AL was dissolved in DMSO, and stored as small aliquots at -20°C. Recombinant human VEGF_165_ was purchased from R&D Systems (San Diego, CA). Antibodies against VEGFR2, STAT3, c-Myc, Bcl-xL, Anti-VEGF, β-actin and phospho-specific anti-VEGFR2 (Tyr^1175^) and anti-STAT3 (Tyr^705^) were obtained from Santa Cruz Biotechnology (Santa Cruz, CA).

### Cell culture

Human umbilical vascular endothelial cells (HUVECs) were from Sciencell (Carlsbad, CA, USA) and cultured in M199 (Invitrogen, Carlsbad, CA) supplemented with 20% fetal bovine serum (FBS). HUVECs were used within passages three to six. Human colorectal cancer cell lines (SW620, HCT116) were purchased from the China Center for Type Culture Collection (Shanghai, China). The cells were cultured according to the supplier’s instruction, at 37°C, 5% CO_2_.

### Cell viability assay

Briefly, HUVECs (5 × 10^3^ cell/well) were seeded onto 0.1% gelatin coated 96-well plates and allowed to attach overnight. After being starved for 7–9 h in M199 containing 1% FBS, the cells were exposed to various concentrations of AL (1, 5, 10, 20 μmol/L ) with or without VEGF (50 ng/mL) for 72 h. Human colorectal cancer cell lines SW-620 and HCT-116 (4.5 × 10^3^ cells/well) were directly treated with AL, respectively. Cell viability was measured by MTT assay. The number of viable cells in treated wells relative to those in control wells gave the percentage of inhibition. Experiments were done in triplicate.

### Endothelial cell migration assay

The migratory activity of HUVECs was assessed using the scratch assay, as previously reported [23]. Briefly, a narrow area on the confluent HUVECs monolayers in 6-well plates were scratched off with a p200 pipette tip. After washing, cells were treated with indicated concentrations of AL in M199 with 1% FBS and 50 ng/mL VEGF. Cells were allowed to migrate for additional 8–9 h, photos were taken from the same areas as those recorded at zero time and the numbers of the migrated cells were counted.

### Transwell invasion assay

The *in vitro* cell invasion assay was performed in the 24-well plates by using a modified Boyden chamber inserts (8 μm) as described previously with modifications [24].The filter membranes were coated with Matrigel (BD Biosciences, San Jose, CA). A single-cell suspension (200 μl serum-free M199 media with 1% FBS) containing 5 × 10^4^ endothelial cells were treated with AL (1, 5, 10, 20 μmol/L) and loaded into the upper chamber. A 500 μL culture medium (1% FBS, 50 ng/mL VEGF) was added to the lower wells of the chamber. After incubation for 8h at 37°C in 5% CO_2_, the migrated cells were fixed and stained with 0.1% crystal violet. Invasiveness was determined by counting the cells that have migrated through the filter. Experiments were performed in triplicates.

### Matrigel tube formation assay

HUVECs were harvested with trypsin, resuspended in 300 μl basic endothelial cell culture medium at a density of 5 × 10^4^ per well and pretreated with AL (1, 5, 10, 20 μmol/L) for 1h with or without VEGF(50 ng/mL) before plating onto the 48-well unpolymerized Matrigel-coated plates. After approximately 9–11 hours of incubation at 37°C in 5% CO_2,_ tube formation was photographed and quantitatively analyzed in randomly chosen microscopic fields (Nikon; original magnification, ×40), by counting the number of tube-like structures formed by connecting endothelial cells. The data presented represent the average of triplicate experiments.

### Live/Dead assay

Apoptosis of cells was also determined by Live/Dead assay (Invitrogen), which was used to measure intracellular esterase activity and plasma membrane integrity as described elsewhere [15].

### Western blotting analysis

To determine molecular mechanism of AL on VEGF-dependent angiogenesis signaling pathway, western blot analysis was performed to detect key proteins involved in the biological functions of endothelial cells and cancer cells. HUVECs were first starved in serum-free ECM for 7 ~ 9 h and then treated with various concentrations AL as indicated in the figures, followed by stimulation with 50 ng/mL of VEGF for 5 ~ 20 min. However, tumor cells were exposed to AL for different duration under the normal culturing conditions. Total cell lysates preparation and Western blot analysis were performed according to the procedure described before [25]. In brief, equal amounts of protein (40 μg) were resolved on (6%-12%) SDS-PAGE, electro transferred onto PVDF membranes, probed with specific antibodies and then detected by chemiluminescence system detection kit (Cell Signaling, Beverly, MA).

### Subcutaneously Xenografted mouse model

All animal experiments were carried out in accordance with a protocol approved by the Institutional Animal Care and Use Committee (IACUC). Briefly, 4 × 10^6^ cells SW620 cells were implanted to 6-wk-old male athymic nude mice in the right flank region. After tumors (100–150 mm^3^) had established, the mice were randomly assigned into two treatment groups containing control and 20 mg/kg of 6 individuals in each by daily oral treatment of AL for consecutive 27 days. The mice of control group were administrated with same amount of pure refined corn oil. Tumor volume was determined by measuring the major (L) and minor (W) diameter with a caliper, and calculated in length × (width^2^)/2. The tumors were excised and weighed after termination of experiments.

### Histology and immunohistochemistry

Tumor were removed and processed for paraffin embedding. Immunohistochemical analysis with anti-CD31 antibody and *in situ* terminal deoxynucleotidyl transferase dUTP nick end labeling (TUNEL) staining were applied on the 5-μm sections. Images were taken using a Leica DM 4000B photo microscope (Solms, Germany; magnification, 400×).

### Statistical analysis

All results are expressed as the mean ± s.d. **S**tatistically differences between the samples were examined by two-tailed Student’s test. A *P* value <0.05 was considered to be statistically significant.

## Results and discussion

### Effect of AL on endothelial cells proliferation *in vitro*

Angiogenesis has been an attractive target for drug therapy because of its key role in the growth and metastatic spread of malignant tumor [26]. An extensive array of nature compounds, particularly those present in dietary and medical plants, have been found to be effective at inhibiting angiogenesis and cancer cell viability, currently in preclinical development [27,28]. Aloe plant, one traditional Chinese medicine, is generally regarded as a safe dietary supplement to treat multiple disorders. Aloin, being a natural compound and the main ingredient of aloe, has been documented for its remarkable potential therapeutic options in cancer. However, its roles in tumor angiogenesis and the involved molecular mechanism are unknown.

To systematically address the contribution of suppressing tumor angiogenesis *in vitro*, we first evaluated the ability of AL to inhibit the proliferation of HUVECs *via* the MTT assay. As shown in Figure 1B, VEGF (50 ng/mL) stimulation increased the numbers of HUVECs ~3-fold. AL at a range of concentrations remarkably decreased VEGF-induced cell viability with the half maximal inhibitory effect at 10 μmol/L in HUVECs when compared with VEGF stimulation alone after 72 h treatment. However, these properties were not due to cytotoxicity of AL in HUVECs, because AL did not show any significant cytotoxic effect on HUVECs at dose up to 40 μM under normal culture conditions (Figure 1C).

### Effect of AL on antiangiogenic function *in vitro*

**Figure 2:**
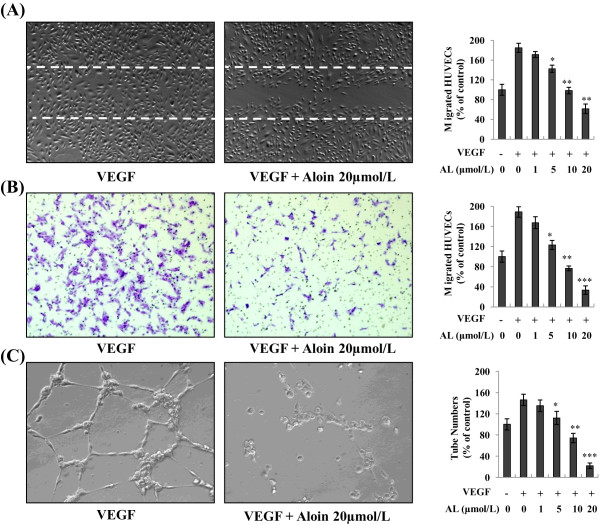
**Effect of AL on VEGF-induced endothelial migration, invasion and tube formation *in vitro*.** HUVECs were plated to full confluence on six-well plates. A single scratch was made and cells were treated with VEGF in the presence or absence of AL. **A**, VEGF stimulation led to an increase in cell migration after 7 h. AL remarkably reduced numbers of migrated endothelial cells induced by VEGF. The migrated cells were quantified by direct counting. **B**, AL inhibited VEGF-induce invasion of HUVECs. Cells were seeded in the upper chamber of Transwell and treated with different concentrations of AL. Representative images were shown as described in “Materials and methods.” **C**, tube formation assay on Matrigel. HUVECs were exposed to different concentration of AL with or without VEGF(50 ng/mL) for 9–10 h. Significant inhibition of endothelial tubular structures formation was observed in a dose dependent manner. Indexing was performed by counting micro tubes or cell in randomly selected four different fields. The results shown are representative of four independent experiments.*, *P* < 0.05; **, *P* < 0.01; ***, *P* < 0.001 *vs.* control.

Chemotactic motility of vascular endothelial cells are important in the angiogenic sprouting process. To determine the effects of AL on endothelial cell migration stimulated by VEGF, we scraped confluent monolayers of HUVECs to clear space for motile cells to move into. As shown in Figure 2A, stimulation by VEGF (50 ng/mL) increased HUVEC motility to nearly fill in the gap after 8 hours of “wounding” the monolayer, however, AL dose-dependently inhibited VEGF-induced migration of HUVECs, with maximal inhibition concentration at 20 μmol/L. The similar effects of AL on the invasive potential of HUVECs were confirmed by the modified Boyden chamber assay, which required cells to degrade and migrate through a sheet to extracellular matrix on a Matrigel-coated membrane. Results showed that VEGF (50 ng/mL) significantly induced a 2-fold increase of endothelial cells invasion *in vitro* (Figure 2B), and this effect was markedly impaired by AL in dose-independent manner (Figure 2B).

We also further evaluated the effect of AL on capillary differentiation of HUVECs on a layer of Matrigel, focusing on the concentration range of 1 to 20 μmol/L. As shown in Figure 2C, endothelial cells differentiate and align to form a highly branched network of capillary-like structures in HUVECs after planted 9–10 h late, while AL treatment (20 μmol/L) caused a significant blockage of the endothelial tubular structures formation (Figure 2C). Quantitative analyses revealed such inhibitory effects of AL were concentration-dependent.

### Effect of AL on signaling pathway in HUVECs

**Figure 3:**
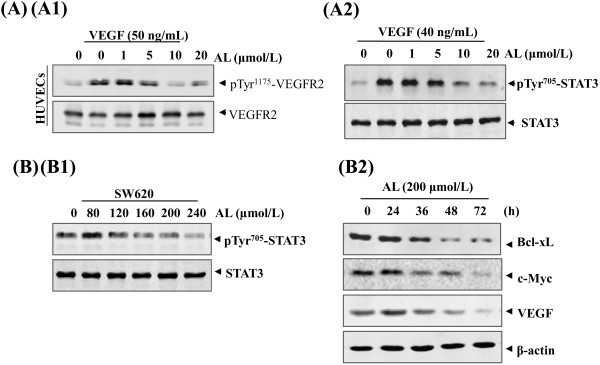
**Effect of AL on STAT3 Pathway in both HUVECs and SW620 cancer cells. A(A1)**, AL suppressed the activation of VEGFR2 triggered by VEGF in endothelial cell. **(A2)**, inhibition pospho-VEGFR2 resulted in a diminished activation of STAT3 in endothelial cells in dose dependent manner. HUVECs were first starved in serum-free ECM for 7–9 h and then pretreated with AL at various concentrations for periods with or without VEGF(50 ng/mL) for 5 ~ 20 min. Total VEGFR2 and STAT3 verified equal protein loading by Western blotting, as described in “Material and Methods.” **B(B1)**, AL suppressed phospho-STAT3 levels in SW620 cell line in a dose manner. Tumor cells were directly treated with the indicated concentration of AL for different duration. The same blots were stripped and reprobed with STAT3 antibody to verify equal protein loading. **(B2)**, AL(200 μmmol/L) suppresses STAT3-regulated genes products (Bcl-xL c-Myc and VEGF) in SW620 cells in time-dependently. β-actin was used as an corresponding internal control to show equal protein loading.

Because the STAT3 pathway plays an important role in cell growth, proliferation, angiogenesis and metastasis et al., we hypothesized AL exhibits anti-angiogenenic activity through blocking STAT3 signaling and next investigated the effects of AL on the key signaling molecules. Whole-cell extracts of VEGF-stimulated cells were analyzed by Western blotting. On ligand stimulation, a marked increase in phosphorylated STAT3 was observed, indicative of receptor activation. Moreover, AL dependently inhibited the VEGF-induced phosphorylation of STAT3 at concentration with maximum inhibition occurring at 10 μmol/L to 20 μmol/L around 3 h (Figure 3A2). The expression of total STAT3 protein was not altered by the drug treatment. In parallel, a rapid down-regulation of VEGFR2 phosphorylation at Tyr 1175 site induced by VEGF (Figure 3A1) was also verified. The levels of total VEGFR2 kinase remained constant under the same conditions (Figure 3A).

Previous studies have indicated that nonreceptor protein tyrosine kinases including JAK2 and Src cooperate to mediate constitutive activation of STAT3 [13,14]. Although this study did not completely demonstrate the effect of AL on JAK2 and Src, we anticipate that the deactivation of STAT3 signaling cascade through suppressing the activation of VEGFR2-mediated c-Src and JAK2 by AL may contribute to tumor angiogenesis inhibition of colorectal cancer.

### Effect of AL on signaling pathway in Colorectal Cancer Lines

To determine whether inhibiting the activation of STAT3 would have a direct antineoplastic activity on cancer cells, we next tested its effect of AL on constitutive STAT3 phosphorylation in SW620 cancer cells. Treatments with AL at indicated concentrations were found to induce down-regulation of phospho-STAT3 in dose-dependent manner but had no impact on total STAT3 expression (Figure 3B1).

As previously mentioned, the STAT3 signaling has been identified to be important in cell survival, proliferation and apoptosis escape of numerous cancers [14].We further investigated whether the expression of STAT3-regulated target gene products was modulated by AL in SW620 cells for various time periods. As the results here presented, three key antiapoptotic (Bcl-xL), pro-proliferation (c-Myc) and angiogenic genes (VEGF) were significantly reduced in response to AL (200 μmol/L), with maximum suppression observed at around 48 to 72 h (Figure 3B2).

Bcl-xL has been reported to block cell death induced by a variety of chemotherapeutic agents [29,30] and commonly confers chemoresistance [31]. Thus, down-regulation of the levels of antiapoptotic (Bcl-xL) and proliferative (c-Myc) proteins products are likely linked with AL’s ability to induce apoptosis, proliferation inhibition and cell cycle arrest in tumor cells. Our findings reported here are similar with previous studies [16,17,20]. In addition, our results also showed that AL treatment could inhibit the secretion of VEGF by cancer cells. VEGF, is one of the most important pro-angiogenic cytokines known and well characterized inducers in tumor neovascularization. The course of the down-regulation of apoptosis- and angiogenesis-related genes by AL might be explained through blocking STAT3 signaling pathway induced a positive feedback loop between angiogenesis and tumor growth.

### Effect of AL on cell viability inhibition and apoptosis induction of colorectal cancer cell lines *in vitro*

**Figure 4:**
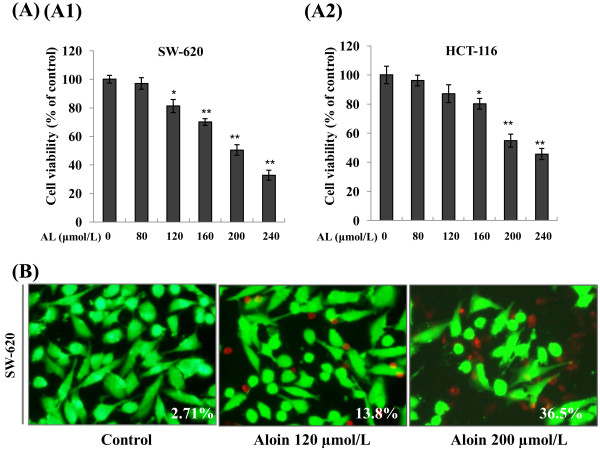
**Effect of AL on proliferation inhibition and apoptosis induction in colorectal cancer *in vitro*. A**, AL significantly suppressed the proliferation of both SW620 and HCT116 cell lines evaluated by MTT assay as described under “Materials and methods”. **B**, Degree of AL-induced apoptosis in SW620 cell lines were determined by Live/Dead assay. Data are presented as relative increase in apoptosis of treated cells compared with untreated control cells. Similar results were obtained in three independent experiments.*, *P* < 0.05; **, *P* < 0.01 *vs.* control.

Because AL treatment affected the activation of STAT3 and STAT3-regulated gene products important for cell survival and apoptosis, we next assessed whether it inhibited the proliferation of tumor cells. Following a 72 h exposure to AL, two CRC cell lines displayed similar sensitivity. As shown in Figure 4A, AL exhibited cell viability suppression on SW620 (Figure 4A1) and HCT116 cells (Figure 4A2) *in vitro*, with an IC_50_ values ranging from 200 μmol/L-240 μmol/L. When we further employed Live-Dead staining assay, our results showed that there was a marked increase in apoptosis as compared to control in a dose-dependent manner. The proportion of apoptotic cells were accordingly increased from 2.71% to 36.5% in SW-620 cells after 72 h exposure to AL (120 μM, 200 μM) (Figure 4B1).

In current study, our data showed that AL significantly inhibited *in vitro* VEGF-induced angiogenic response of human endothelial cells, to inhibit proliferation and migration of endothelial cells, and to reduce the ability to form capillary vessel, with maximum inhibition dose observed at 10 μmol/L to 20 μmol/L. When compared the effective concentrations of AL on endothelial cells (Figures 1 and 2, 10 ~ 20 μmol/L) and colorectal tumor cells (Figure 4A, 200 ~ 240 μmol/L), we found that AL might conceivably affect tumor-induced angiogenesis *in vitro* at local concentration much lower than those necessary to cause a cytotoxic effect on cancer cells, indicating that AL is more effective in angiogenesis disease condition. The anti-angiogenesis mediated by AL on endothelial cells may be earlier than a direct cytotoxic effect on tumor cells.

### The antitumor effects of AL *in vivo*

**Figure 5:**
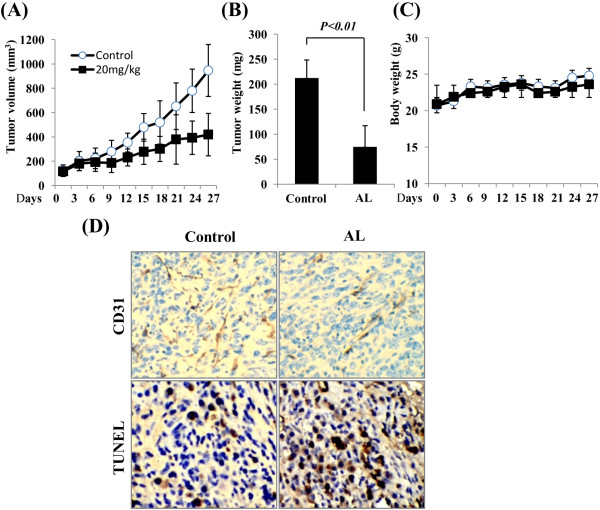
**Effect of AL on tumor growth arrest and angiogenesis inhibition in SW620-bearing mice.** Daily oral treatment with AL at dosages of 20 mg/kg was initiated when tumor volumes reached approximately 120 mm^3^, as described in “Materials and methods.” **A-C**, AL resulted in tumor growth inhibition of 63% at day 27 as measured by tumor volume and weight with little toxicity at the tested dose. Same amount of pure refined corn oil served as vehicle controls. **D**, Immunohistochemical and TUNEL analysis showed that AL inhibited numbers of CD31-positive blood vessels and induced apoptosis in human colorectal cancer xenografts. Original magnification, ×400. **, *P* < 0.01 *vs.* control.

In the present study, human SW620 CRC nude mouse xenograft model was well performed to validate our results *in vitro*. As shown in Figure 5A and B, treatment of SW620 tumor-bearing animals (n = 6) at 20 mg/kg/d body weight, oral gavage, once daily, resulted in growth inhibition of 63% at day 27. There was no significant weight loss (Figure 5C) or other signs of acute or delayed toxicity (data not shown) compared to controls, indicating little toxicity response for AL. In our experiment system, both high and low dosages of AL were also tested; however, 10 mg/kg of AL did not effectively inhibit tumor volume and 30 mg/kg of AL showed some toxic effect on the body weight of mice.

We next performed immunohistochemistry with anti-CD31 antibody and TUNEL analysis on tumor sections from xenografted mice with or without the treatment of AL. Immunostaining revealed large numbers of CD31-positive blood vessels throughout the tumor of untreated mice, whereas fewer CD31-positive vessels were found in AL-treated tumors (Figure 5D, left). Additionally, apoptotic cells were increased in AL-treated group as indicated by TUNEL analysis (Figure 5D, right). Consistent with previous results observed *in vitro and in vivo*, our data supported our hypothesis that AL can cooperate to suppress tumor growth of human colorectal cancer xenografts by exerting a primary anti-angiogenic activity on endothelial cells at lower concentrations (below 20 μM) and a direct apoptotic effect on tumor cells at higher concentrations (up to 200 μM) through STAT3 signaling pathway.

## Conclusion

AL may represents one safe, affordable and orally active drug applied in clinical practice for cancer prevention and therapy, even at high doses. In the future, experimental as well as clinical studies e.g. regarding the combination of AL and conventional chemotherapeutics will further elucidate its therapeutic value in human colorectal cancer.

## Competing interests

The authors declare that they have no competing interests.

## Authors’ contributions

TL designed the research. PQ performed the experiments throughout this research. PHM participated in its design and coordination. LHZ analyzed the data; XYH contributed to the writing of manuscript. All authors have read and approved the final manuscript.
